# The association between dengue immunoglobulin G titres with previous clinical dengue infection and white cell counts in Cuban children: A population-based study

**DOI:** 10.1371/journal.pone.0207391

**Published:** 2018-11-28

**Authors:** Ramón Suárez-Medina, Silvia Josefina Venero-Fernández, Lourdes Batista-Gutierrez, Yanelis de los Angeles Estrada-Rondon, Anadelis Alfonso-Hernandez, Dulcima Casanave-Guarnaluce, Nieves Sardinas-Baez, Ivette Castillo-Aguilar, Jorge Antonio Febles-del Toro, Andrew W. Fogarty, the HINASIC (Historia Natural de la Sibilancia en Cuba/Natural History of Wheezing in Cuba) Study Group

**Affiliations:** 1 Instituto Nacional de Higiene, Epidemiología y Microbiología, Infanta No 1158 e/ Llinás y Clavel, Código Postal, La Habana, Cuba; 2 Dirección Municipal de Salud Pública municipios Cerro y Arroyo Naranjo, La Habana, Cuba; 3 Nottingham NIHR Biomedical Research Unit, Division of Epidemiology and Public Health, University of Nottingham, Clinical Sciences Building, City Hospital, Nottingham, United Kingdom; University of Malaya, MALAYSIA

## Abstract

**Background:**

The prevalence of dengue infection is increasing globally. There are few prospective population-based surveillance studies of the immunological and inflammatory consequences of exposure to dengue virus in young children.

**Objective:**

To study the association between serologically confirmed prior medical diagnosis of dengue infection and blood measures of systemic inflammation with dengue virus immunoglobulin G levels.

**Methods:**

A population-based study of healthy three-year old children living in Havana, Cuba.

**Results:**

865 individuals provided a blood sample. Fourteen (1.6%) had a prior medical diagnosis of dengue infection, and 851 individuals had no prior medical diagnosis. There was no difference in the serum immunoglobulin G titres between these groups (Mann-Whitney test, p = 0.49). Total white cell count, blood neutrophil and eosinophil counts were linearly associated with a dengue immunoglobulin G value above the median value.

**Conclusions:**

There was no difference between the dengue immunoglobulin G titres in young children who had previously had clinically proven dengue infection compared to those who had no diagnosis of prior infection. This may be a consequence of a relatively high prevalence of sub-clinical infection. A higher dengue immunoglobulin G level was positively associated with a range of inflammatory biomarkers, although these data cannot demonstrate a causal association.

## Introduction

Although dengue infection results in substantial morbidity and mortality [1], there have been no population-based studies in young children aged three years, and as a consequence there are no clear definitions of dengue immunoglobulin G (IgG) serological thresholds to define prior exposure. However, knowledge of cases of definite prior clinical infection with dengue virus may help identify these threshold values, and also permit comparison with a control group with no prior infection, hence allowing estimation of the number of sub-clinical cases of dengue infection.

We have used data from a community-based study of healthy three-year old children living in Havana, Cuba, to explore the association of increased titres of dengue IgG immunoglobulin for those with a definite prior clinical diagnosis of dengue infection, compared to those with no history of dengue infection. We also assessed the association of increased titres of dengue IgG immunoglobulin with blood measurements of inflammation as previous studies have demonstrated that leucocytes are decreased during acute dengue virus infection [2–9].

## Methods

### Study population

The study population was originally selected to investigate the risk factors for asthma and allergic disease for children living in Havana, Cuba and has been described in detail previously [10]. Briefly, 1956 children aged 12 to 15 months were randomly selected from four municipalities across Havana in 2010 and 2011 and data collected on their family history and living environments. Parents or guardians of all children who participated gave written consent, and the study was approved by both the Instituto Nacional de Higiene, Epidemiología y Microbiología Ethics Committee (Havana, Cuba) and the University of Nottingham Ethics Committee (Nottingham, UK).

### Data collection

Baseline data were collected in the first year of the study. The study participants were subsequently followed up each year and the third year of data collection spanned June 2012 to July 2013. At the time of the follow-up interview in the third year of data collection the parent/guardian was asked if and when the child had been diagnosed with dengue infection by the Cuban health authorities. All children with suspected dengue infection in Cuba are referred to the hospital for investigation including serological (IgM) testing and these results are then given to the parents/guardian. A blood sample was collected from children who had no current symptoms of viral infection and this was stored in a freezer at -20 degrees Centigrade. This was subsequently defrosted and analysed for dengue immunoglobulin G (IgG) serology using the Vircell assay to generate an antibody index [11], serum immunoglobulin E (IgE) [12], highly sensitive C- Reactive Protein (hsCRP) [13]. A full blood count allowed the haemoglobin level and circulating white cell count to be measured.

### Statistical analysis

**Fig 1:**
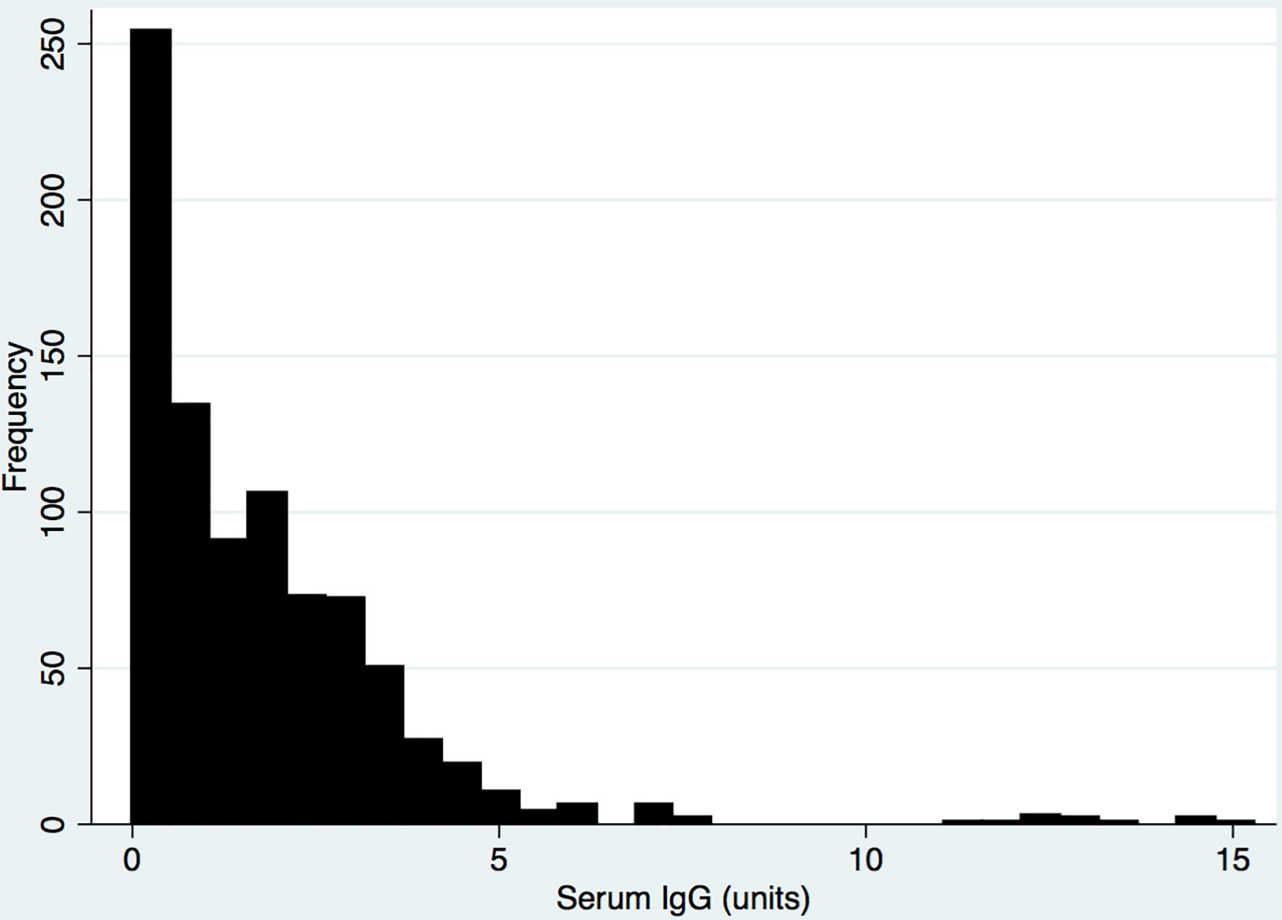
Histogram of dengue IgG levels.

The cases with a definite prior medical diagnosis of dengue infection were compared with those with no prior medical diagnosis of dengue infection. The exposure of dengue IgG levels were initially plotted as a histogram to explore the distribution. This demonstrated that a substantial proportion of the population has no serum dengue IgG response (Fig 1). A binary outcome measure of an elevated serum IgG titre was then generated by calculating the median value. An elevated dengue IgG was defined as above the median value, and compared with those values that were equal to or below the median.

Logistic regression was used to explore the association of the blood exposures of interest with an increased dengue IgG titre with the various outcome measures using Stata v14 statistical software (Texas, USA).

## Results

1543 individuals provided data for the third year of the study, and of these 865 (56%) donated a blood sample that was analysed to generate the dengue IgG measurements (Table 1). 439 individuals (51%) were male and the median age was 39.6 months (interquartile range 37.2 to 42.0). The range of dengue IgG titres for the study population is presented in the Fig 1, and had a median value of 1.3 (interquartile range of 0.37 to 2.6).

**Table 1:** Study population with associations with increased dengue IgG titres.

	Summary statistics	Association with increased dengue levels* (95% CI)
Females Males	426 (49%) 439 (51%)	1.00 0.93 (0.74 to 1.17)
Median age, months	39.6 (IQR 37.2 to 42.0)	1.03 (1.02 to 1.04)
Mean total white cell count, x10^3^/μL	8.7 (sd 1.7) N = 793	0.87 (0.78 to 0.96)
Mean neutrophil count, x10^3^/μL	3.8 (1.5) N = 792	0.90 (0.85 to 0.95)
Mean eosinophil count, x10^3^/μL	0.4 (0.4) N = 783	0.70 (0.61 to 0.78)
Mean lymphocyte count, x10^3^/μL	4.4 (1.4) N = 792	0.94 (0.82 to 1.07)
Log serum IgE, IU/ml	3.8 (1.3) N = 850	0.95 (0.86 to 1.04)
Log serum hsCRP, mg/L	-1.9 (2.7) N = 850	0.98 (0.93 to 1.04)

Statistical analysis using logistic regression with robust standard errors to adjust for clustering by municipality. sd = Standard deviation. CI = confidence intervals. ISR = interquartile range

* odds ratio of risk of having serum dengue titre above the median value

**Fig 2:**
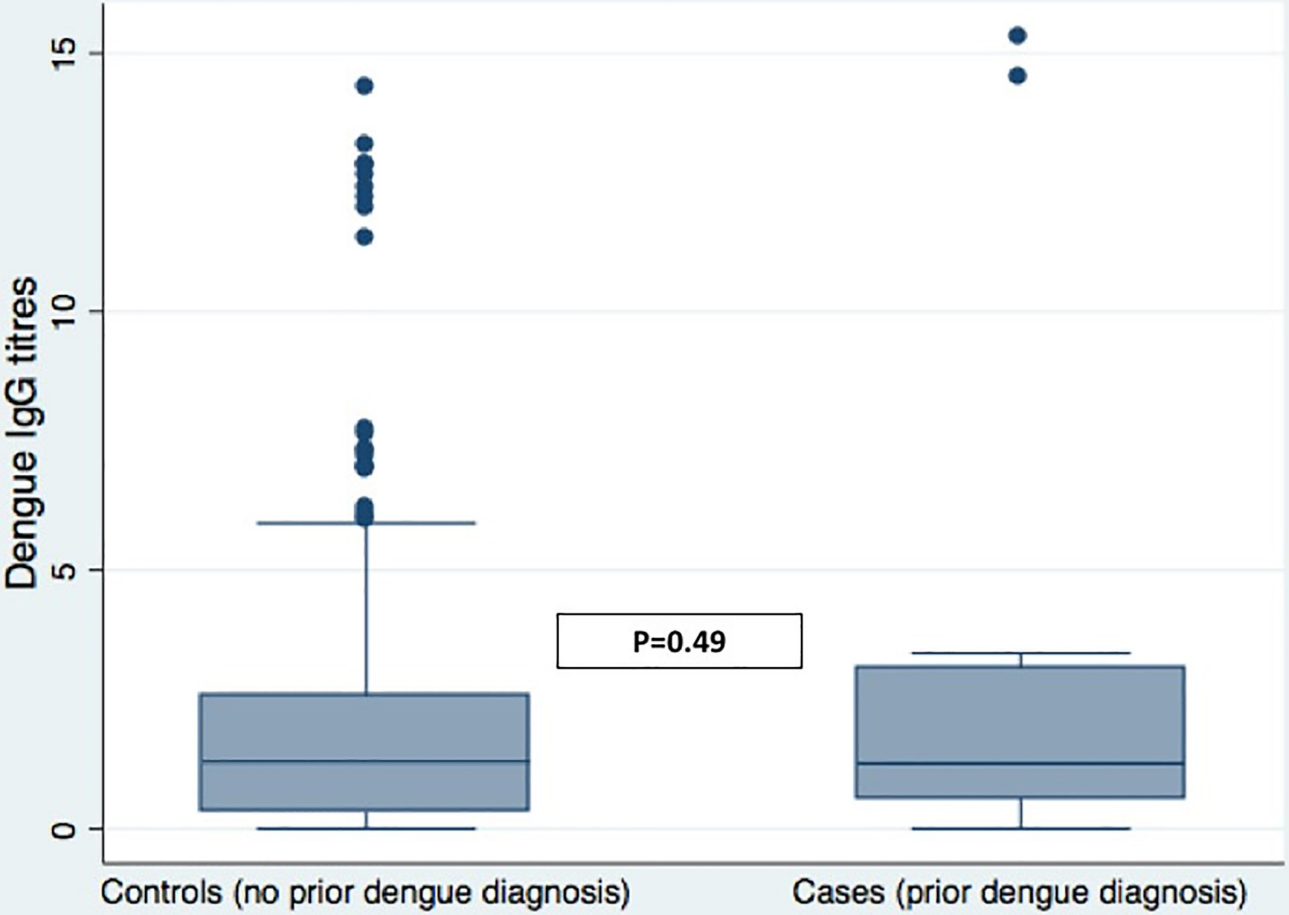
Comparison of the distribution of IgG dengue serology stratified by cases with medically confirmed dengue infection and controls with no medical diagnosis of dengue infection. The box plot shows the median value (central line in box) and the 25^th^ and 75^th^ percentiles (horizontal lines of box). The individual horizontal lines demonstrate the limits of adjacent data, and the dots represent the outlying values. Statistical comparison used the Mann-Whitney test.

There were 14 (1.6%) individuals with a prior medical diagnosis of dengue infection, and these cases had a median dengue IgG titre of 1.26 (interquartile range IQR 0.60 to 3.10). There were 851 individuals with no prior medical diagnosis of dengue infection, and this group had a median IgG titre of 1.30 (0.34 to 2.57). There was no statistical difference between the IgG levels for these two groups (Fig 2, Mann-Whitney test, p = 0.49, S1 Data). Four hundred and thirty one individuals (50.6%) with no previous medical diagnosis of dengue infection had IgG titres above the median value for those who had a prior medical diagnosis of dengue infection, while only 131 individuals (15.4%) of this group had no dengue IgG response at all.

The total white cell count was linearly negatively associated with a dengue IgG value above the median value with an odds ratio (OR) of 0.87 (95% confidence intervals CI: 0.78 to 0.96) per unit increase in white cell count. Blood neutrophil and eosinophil counts were also linearly negatively associated with dengue IgG, with ORs of 0.90 (95% CI: 0.85 to 0.95) and 0.70 (95% CI: 0.61 to 0.78) respectively. There was no association of blood lymphocyte counts, log serum IgE, or log serum hsCRP with increased blood dengue IgG levels

## Discussions

This is the first population-based study in three-year old children to explore the differential serological response between cases with medically confirmed prior infection and those with no previous infection. There was no significant difference between the two groups, and there was a substantial proportion of the non-infected control group with IgG titres above those of the infected group, suggesting possible sub-clinical infection. We also identified that an elevated dengue IgG level was associated with decreased blood leucocyte levels, lower neutrophil and eosinophil levels, although our cross-sectional study design does not allow any causal inferences to be drawn.

The strengths of this analysis include the population-based nature of the cohort, and the use of objective measures of both the exposures and the outcome measures to minimise the risk of observer bias. The study was based in policlinics that are the first point of contact for Cubans with the national health system. The study was designed to study allergic disease and hence there was no sensitisation or risk of increased awareness of the risk of dengue infection above that expected in the general Cuban population, who were experiencing an increase in the incidence of dengue infection cases at the time [14]. All children with suspected dengue infection in Cuba are referred to the hospital for investigation including serological (IgM) testing, and hence we are confident that a positive medical diagnosis of dengue is correct. In addition, the absence of alternative health providers in Cuba makes it unlikely that a diagnosis of acute dengue infection would be made elsewhere. As the dengue IgG assay that we used has not been validated as a measure of previous infection in three-year old children, a binary outcome variable was generated based on whether an individual’s dengue IgG response was above or below the median value for the whole population. This is a pragmatic but relatively simple statistical test that demonstrates that it is unlikely that these associations occurred by chance, but do not explain the natural history of any biological processes that may be involved. It is well recognised that the antibody response to external exposures in young children below the age of five years old is limited, increasing as the child gets older [15], and hence this binary outcome measure of dengue IgG may not necessarily equate to prior clinical infection [11]. It is important to clarify that our data may not be generalizable to other different populations, especially those that are older and hence are likely to have more mature immune systems, or that are living different in other geographical locations where the prevalence of dengue and other associated environmental factors will differ to those in Cuba.

The comparison of the serological IgG titres between the diagnosed cases of prior dengue infection and the control group with no prior infection is interesting. There is no difference between the serum IgG levels between the two groups, and although the number of cases is relatively low, we would anticipate that these numbers would be sufficient to observe a difference in serum dengue IgG titres if the control group has had no prior exposure at all to dengue virus. This raises the possibility that the control group have had substantial sub-clinical dengue virus exposure. Quantifying this population is challenging in the absence of a threshold for the IgG serological assay, but 51% of the control population had a serum IgG level above the median value of those who have had medically confirmed dengue infection and 15% had no detectable serum IgG to dengue. An alternative explanation for the absence of a difference between IgG titres between the two groups is that in the context of a maturing immune system, some of the children who had definite prior IgG infection were not able to generate a robust immune response. However, as age was positively associated with risk of increased dengue IgG and is also a measure of the duration of risk of exposure to dengue infection, the former explanation is more plausible.

The observation that dengue virus exposure is often a sub-clinical event has been made previously in a large epidemiological study of children and adults living in Santiago de Cuba, in 1997. This analysis estimated that approximately 3% of primary dengue infections were clinically overt, with the remaining 97% being subclinical [16]. Some of these subclinical cases may constitute children who experienced a low-grade febrile episode that was independently managed by the carers, and did not come to the attention of the medical authorities. Infection with a variety of viruses including arboviruses are present in the tropics, and are understandably not always are bought to the attention of the medical authorities. Our data are consistent with this hypothesis, and suggest that there is no significant difference in the residual IgG immune response between those who had clinically overt dengue infection and those who did not. The immune response may be of potential clinical importance, as a secondary dengue infection after a primary infection generates a more symptomatic dengue infection [17, 18]. This is an area with limited epidemiological data, and hence continued observation of similar cohorts with good baseline data will help increase understanding of the natural history of clinical response dengue infection over the life course.

In conclusion, data from a population-based study of healthy young children living in Havana, Cuba has demonstrated that cases with prior serologically confirmed dengue infection have similar dengue serological IgG levels to those with no prior medical diagnosis of dengue infection. This may represent a substantial prevalence of sub-clinical dengue infection.

## Supporting information

S1 Data The dengue serology data can be found at plosonedenguedata. (XLS)
